# Factors Associated with Symptoms of Depression and Psychological Distress during the COVID-19 Pandemic

**DOI:** 10.3390/bs11020013

**Published:** 2021-01-21

**Authors:** Yuqi Guo, Omar T. Sims, Weidi Qin, Fan Yang

**Affiliations:** 1 School of Social Work, College of Health and Human Services, University of North Carolina Charlotte, Charlotte, NC 28223, USA; yguo16@uncc.edu; 2 Department of Social Work, College of Arts and Sciences, University of Alabama at Birmingham, Birmingham, AL 35294, USA; 3 Department of Health Behavior, School of Public Health, University of Alabama at Birmingham, Birmingham, AL 35294, USA; 4 Center for AIDS Research, School of Medicine, University of Alabama at Birmingham, Birmingham, AL 35294, USA; 5 Integrative Center for Aging Research, School of Medicine, University of Alabama at Birmingham, Birmingham, AL 35294, USA; 6 Jack, Joseph and Morton Mandel School of Applied Social Sciences, Case Western Reserve University, Cleveland, OH 44106, USA; wxq32@case.edu; 7 Social Welfare Program, School of Public Administration, Dongbei University of Finance and Economics, Dalian 102488, China; 8 Institute of Quantitative & Technological Economies, Chinese Academy of Social Sciences, Beijing 102488, China

**Keywords:** COVID-19, depression, psychological distress, China, social distancing, social media

## Abstract

The objective of this study was to examine factors associated with symptoms of depression and psychological distress during the COVID-19 pandemic in China. Convenience sampling and snowball sampling were used to recruit a sample of adults in China (*n* = 2130) from 14 February 2020 to 3 March 2020 to complete an online survey. Linear regression was used to examine the predictors of symptoms of depression and psychological distress. Living in a non-urban area and the number of confirmed cases in their city of residence were positively associated with symptoms of depression. Female gender, not being married, practicing social distancing, the amount of time spent daily on social media searching for and reading information on COVID-19, the number of confirmed cases of COVID-19 in their city of residence, and having confirmed or suspected cases of COVID-19 in personal networks were positively associated with psychological distress. Social distancing is a widely used public health approach for population-wide virus-containment of COVID-19. However, reductions in population-wide psychological well-being are inadvertent consequences of social distancing. There is an emerging need to negate factors that increase adverse mental health vulnerabilities during the COVID-19 pandemic.

## 1. Introduction

The novel coronavirus disease 2019 (COVID-19) emerged as a cluster of unexplained cases of pneumonia in Wuhan, China but rapidly spread throughout the world and emerged as a global pandemic [1]. As of 14 December 2020, China had 95,064 confirmed cases of COVID-19 and 4758 confirmed deaths related to COVID-19 [2]. Worldwide, there were 71,051,805 confirmed cases of COVID-19 and 1,608,648 confirmed deaths related to COVID-19 [2]. 

COVID-19′s R^0^ of 5.7 (i.e., the average number of people who will acquire an infection from one person with COVID-19) [3] has hastened the global pandemic, led to a massive international public health crisis, and hindered public health efforts to control the pandemic. Those infected with COVID-19 experience a wide range of symptoms including but not limited to fever, cough, shortness of breath, fatigue, muscle or body aches, and new loss of taste or smell [4]. Chronic kidney disease, hypertension, chronic obstructive pulmonary disease (COPD), obesity, type 2 diabetes mellitus (T2DM), and primary immunodeficiency diseases (PIDs) are a few underlying medical conditions associated with elevated risks for COVID-19 disease severity and death [5,6].

An infectious disease pandemic can lead to an iatrogenic pandemic of panic or pandemic-related psychological distress in the general population often characterized by fear, nervousness, fright, excessive worry, and other anxiety and panic behavior, and the recent COVID-19 pandemic is no exception [7]. There are many factors related to COVID-19 that can alter and negatively impact psychological well-being in the general population. For example, fear of acquiring the infection, shortage of personal protective equipment (PPE), limited resources for testing and treatment, economic and financial loss, and repeated school and work closures may lead to psychological distress [8,9,10,11].

The first nationwide large-scale survey on psychological distress that was conducted early in the epidemic in China found that nearly 35% of the general population experienced psychological distress due to COVID-19 [12]. In an iterative study a month later, 54% of the general population in China rated the psychological impact of COVID-19 as moderate to severe, 28.8% reported moderate to severe symptoms of anxiety, 16.5% reported moderate to severe symptoms of depression, and 8.1% reported moderate to severe levels of stress [13]. Similarly, a cross-sectional epidemiological study reported that 29% and 37% of Chinese adults experienced symptoms of anxiety and depression, respectively [14]. A recent meta-analysis of 19 studies conducted in China estimated that the prevalence of stress related to the COVID-19 pandemic was 48%, and prevalence estimates of depression and anxiety related to the COVID-19 pandemic were 27% and 22%, respectively [15]. Furthermore, increased symptoms of depression, anxiety, and psychological distress during the COVID-19 pandemic have been observed in several other countries (e.g., Italy, Turkey, and Saudi Arabia) [16,17,18,19].

However, more research on the mental health consequences of COVID-19 is needed. The number of published studies on the biological and clinical consequences of COVID-19 dwarfs the number of studies that provide mental health characterizations and consequences of COVID-19. Equally important, more empirical data on factors associated with symptoms of depression and psychological distress are needed. Given the universality of the COVID-19 pandemic, this knowledge would be advantageous for mental health professionals in and outside of China who provide mental health care and treatment to those in clinical and public health settings. To fill these respective knowledge gaps, the objective of this study is to examine factors associated with symptoms of depression and psychological distress during the COVID-19 pandemic among adults living in China. 

To achieve the study objective, the study specifically considered multi-level factors guided by Bronfenbrenner’s Ecological Systems Theory (EST) [20]. EST is a widely used theoretical model that considers ways in which interrelated and multi-level individual, community, and environmental factors influence health and mental health [21,22]. In particular, EST has previously been used to examine protective factors and risk factors that impact smoking behaviors, weight-related behaviors, obesity, and health-related microaggressions [23,24,25,26]. EST proposes that an individual’s health and mental health status are enmeshed in and affected by a variety of surrounding micro-, exo-, meso-, and macrosystems. The microsystem includes individual level factors (e.g., sex, age, and health); the exosystem includes family level factors (e.g., location and family support); and the mesosystem and macrosystem include indirect environmental level (e.g., neighborhood and community services) and social and culture level factors (e.g., social media), respectively. 

For the current study, individual factors (e.g., age, gender, education, marital status, income, self-rated general health, and social distancing behaviors), community factors (e.g., residential area and living arrangement), and environmental factors (e.g., time spent daily on social media reading searching for and reading information on COVID-19, number of confirmed cases of COVID-19 in their city of residence, and confirmed or suspected cases of COVID-19 in personal networks) were used to guide the analysis. The first hypothesis for this study is that symptoms of depression and psychological distress are elevated during the COVID-19 pandemic. The second hypothesis is that living alone, not practicing social distancing, the amount of time spent daily on social media searching for and reading information on COVID-19, and the number of confirmed cases of COVID-19 in their city of residence are positively associated with increases in symptoms of depression and psychological distress. 

## 2. Materials and Methods

### 2.1. Study Design

Convenience sampling and snowball sampling were used to recruit a sample of Chinese adults (≥18 years old) in China. A recruitment announcement for study participation was emailed on an institutional listserv to those employed by and affiliated with the Dongbei University of Finance and Economics (Dalian, China). The email explained the purpose of the study and that participation was voluntary, and the email provided a weblink to the online survey in Wenjuanxing (the Chinese version of Qualtrics). Similarly, recruitment announcements for study participation were posted on Wechat and Weibo (Chinese versions of Twitter). Those who participated were asked to “retweet” the announcement to others in their social media networks for potential participation. Participants who fully completed survey questions from 14 February 2020 to 3 March 2020 (*n* = 2130) were included in the present study. Participants provided informed consent and were informed that their responses would remain anonymous. The study was conducted according to the guidelines of the Declaration of Helsinki and was approved by the Dongbei University of Finance and Economics. 

### 2.2. Outcomes

*Symptoms of depression and psychological distress*: the primary outcomes of interest were predictors of symptoms of depression and psychological distress. Symptoms of depression include but are not limited to depressed mood most of the day, diminished interest in pleasurable activities, insomnia or hypersomnia, significant weight loss, and fatigue [27]. Psychological distress is a discomforting, emotional state often caused by a specific stressor or demand [28]. The Center for Epidemiologic Studies Depression Scale (CES-D-10) was used to measure symptoms of depression [29,30]. The CES-D-10 is a short, 10-item self-report Likert scale that assesses symptoms of depression in the past week (e.g., felt depressed, felt fearful, felt lonely, and sleep was restless) (0 = “rarely or none of the time” to 4 = “all of the time”). The total score range of the CES-D-10 is 0 to 30. Higher scores on the CES-D-10 suggest greater severity of symptoms of depression, and a score ≥ 10 is indicative of significant symptoms of depression. Psychometric properties of the CES-D-10 have been previously validated for use among Chinese adults [31,32,33]. In the current study, the CES-D-10′s Cronbach’s alpha was 0.83. The Kessler Psychological Distress Scale (K6) was used to measure psychological distress [34]. Using a 5-point Likert scale (1 = “none of the time” to 5 = “all of the time”), the 6-item scale measures psychological distress based on a framework that includes assesses emotional, behavioral, and psychophysiological manifestations (e.g., nervousness, hopelessness, and worthlessness) [35]. Higher scores on the K6 are indicative of higher psychological distress. In the current study, the K6′s Cronbach’s alpha was 0.84.

### 2.3. Predictors

*Individual factors*: the individual factors included self-reported age, gender, education, marital status, income (i.e., CNY ¥0–10,000, 10,001–30,000, 30,001–50,000, 50,001–100,000, and >100,000 in Chinese currency), social distancing, and self-ratings of general health. The following questions, adapted from social distancing recommendations published by the Centers for Disease Control and Prevention (CDC) [36], were used to assess participants’ social distancing behaviors: “Since the COVID-19 outbreak, have you (1) avoided social gatherings, (2) avoided contacting people not living in your household, and (3) self-isolated at home?” Participants who endorsed yes on all three questions were defined as engaging in social distancing. For self-ratings of general health, participants were asked to self-rate their general health (e.g., 0 = poor, 1 = fair, 2 = good, 3 = very good, and 4 = excellent).

*Community factors*: the community factors included self-reported residential area (urban or non-urban area) and living arrangement (lived alone or lived with someone).

*Environmental factors*: the environmental factors included the amount of time spent daily on social media searching for and reading information on COVID-19, the number of confirmed cases of COVID-19 in their city of residence, and confirmed or suspected cases of COVID-19 in personal networks. The following question was used to assess the amount of time spent daily on social media searching for and reading information on COVID-19: “How much time do you spend daily on social media searching for and reading information on COVID-19 (e.g., news coverage, updates, protection knowledge, and online discussions)?” (never to >1 h). The following question was used to ask participants about the number of confirmed cases of COVID-19 in their city of residence: “There are how many confirmed cases of COVID-19 in your city of residence?” (none to >300). Last, the following question was used to ask participants if there were confirmed or suspected cases of COVID-19 in personal networks: “Are there confirmed or suspected cases of COVID in your personal network?” (yes or no). 

### 2.4. Statistical Analysis

Measures of central tendency and frequency distributions were used to characterize the sample. Linear regression, using ordinary least squares (OLS), was used to examine the predictors of symptoms of depression and psychological distress. Because age was positively skewed, age was log transformed for the analysis. The amount of time spent daily on social media searching for and reading information on COVID-19 and the number of confirmed COVID-19 cases in their city of residence were treated as quasi-interval variables in the OLS regression models. Statistical assumptions were checked ahead of running OLS regression models. No violations of normal distribution of continuous variables were detected (skewness ≤ |1.43| and kurtosis ≤ |3|) [37]. Linear regression models were without multicollinearity (variance inflation factors (VIFs) ranged from 0.36 to 0.99) [38]. All statistical analyses were conducted in Stata 14.

## 3. Results

### 3.1. Sample Characteristics

The mean (SD) age of participants was 28.43 (10.46) (Table 1). The majority of participants were female (54.84%), had an education beyond high school (78.45%), were not married (68.64%), and had an income between ¥0 to 10,000 (56.76%). The mean self-rating of general health was 4.35 (0.75) (i.e., very good). The majority of participants practiced social distancing (95.63%) and lived in non-urban area (52.39%). More than one-third (34.04%) spent 16–30 min daily on social media searching for and reading information on COVID-19; more than one-third (39.01%) reported 10–100 confirmed cases of COVID-19 in their city of residence; and a little more than one-tenth (13.29%) reported having confirmed or suspected cases of COVID-19 in personal networks. Mean symptoms of depression (e.g., CES-D-10) and psychological distress (e.g., K6) scores were 14.65 (5.49) and 7.31 (4.71), respectively. 

### 3.2. Multivariate Analysis

#### Predictors of Symptoms of Depression

Living in a non-urban area (*t* = 3.02, *p* = 0.000) and the number of confirmed cases in their city of residence (*t* = 2.01, *p* = 0.04) were positively associated with symptoms of depression (Table 2). Practicing social distancing (*t* = −2.21, *p* = 0.021) was negatively associated with symptoms of depression. 

### 3.3. Predictors of Psychological Distress

Female gender (*t* = 3.09, *p* < 0.00), not being married (*t* = 2.43, *p* = 0.01), practicing social distancing (*t* = 2.73, *p* = 0.00), amount of time spent daily on social media searching for and reading information on COVID-19 (*t* = 3.73, *p* < 0.00), the number of confirmed cases of COVID-19 in their city of residence (*t* = 2.57, *p* = 0.01), and having confirmed or suspected cases of COVID-19 in personal networks (*t* = 3.31, *p* = 0.00) were positively associated with psychological distress (Table 3). An education level greater than high school (*t* = −2.13, *p* = 0.03) was negatively associated with psychological distress. 

## 4. Discussion

This study examined the factors associated with symptoms of depression and psychological distress during the COVID-19 pandemic among adults living in China. Several notable findings emerged from the analysis. First, symptoms of depression and psychological distress were elevated during the COVID-19 pandemic. Higher education was a protective factor against increases in psychological distress, the number of confirmed cases of COVID-19 in city of residence was a risk factor for increases in symptoms of depression and psychological distress, and confirmed or suspected cases of COVID-19 in personal networks was a risk factor for increases in psychological distress [19,39,40]. However, living in a non-urban area was a risk factor for increases in symptoms of depression. The findings suggest that the COVID-19 pandemic, the number of cases of COVID-19, and positivity within personal networks all have broad deleterious effects on psychological well-being, while higher levels of education may be protective of psychological well-being. Similar findings have been found in other studies conducted in Italy [16,39]. On the contrary, living in a non-urban area was not associated with increases in symptoms of depression in a study conducted in Turkey [19]. Living in an urban area was a risk factor for symptoms of depression. That study’s sample was largely urban (81%), whereas a little more than half of the sample from the present study was non-urban (52%). However, the discrepancy in this opposing finding is unclear. Future studies are encouraged to further investigate associations between residential area and depression during the COVID-19 pandemic. Compared to urban areas, non-urban areas typically have greater and more complex issues related to availability and accessibility of local mental health services. It is plausible that the COVID-19 pandemic has further compounded these respective issues in a population with preexisting vulnerabilities. Public health efforts are needed to combat and reduce the psychological consequences of COVID-19 in the general population and to eliminate geographic disparities in psychological well-being within the general population.

Second, social distancing was independently associated with increases in psychological distress. The primary purpose of social distancing is virus-containment, and the primary endpoints are reductions in COVID-19 transmission. Due to the highly contagious nature of COVID-19, social distancing is a public health necessity. However, long durations of social distancing, frustration and boredom with social distancing, avoidance of those who may test positive, limited to no physical contact with social and familial networks, and limited or inadequate access to basic supplies all have the potential to create and increase psychological distress [41,42]. Psychological distress is an unintended aftereffect of social distancing. Public health and clinical intervention models are needed not only to mitigate the mental health consequences of COVID-19 but also to concurrently mitigate the psychological aftereffect of social distancing. Even with the advent of an effective COVID-19 vaccine, it is probable that reliance on virus-containment strategies such as social distancing will continue for the foreseeable future—at least until widespread accessibility and availability of a COVID-19 vaccine and herd immunity are both achieved. The advent of a COVID-19 vaccine may loosen some social distancing parameters, but it will not fully eliminate the need for social distancing or its aftereffects.

Conversely, the association between social distancing and decreases in symptoms of depression was somewhat unexpected—mostly because studies from several countries have demonstrated that the COVID-19 pandemic has led to considerable increases in symptoms of depression [43,44,45,46,47,48]. However, after conducting an in-depth literature search, participant compliance with COVID-19 precautionary measures may explain the association. In a recent and large survey internet-based cohort study (*n* = 1642) conducted in Europe, a higher compliance with recommended precautionary measures to prevent the spread of COVID-19 (e.g., hand hygiene, wearing a mask regardless of the presence or absence of symptoms, covering the mouth when coughing and sneezing, and washing hands immediately after coughing or sneezing) predicted lower depression scores, and similar findings were reported elsewhere [13,49]. Unfortunately, the current study did not assess or include variables related to compliance with COVID-19 precautionary measures, and the social distancing variable was too broad to serve as a proxy. Associations between compliance with COVID-19 precautionary measures and symptoms of depression are understudied, and future studies are encouraged to consider assessment and inclusion of compliance with COVID-19 precautionary measures when examining factors associated with symptoms of depression. If the finding is replicated, iterative studies are encouraged to elucidate the association.

Fourth, the amount of time spent daily on social media searching for and reading information on COVID-19 was associated with increases in psychological distress. A previous study reported that people spend more time on social media during the COVID-19 pandemic compared to social media usage before the pandemic [50] and that people who excessively use social media for COVID-19 information are more likely to experience increases in feelings of fear and in symptoms of anxiety [40,50]. Social media is an easily accessible platform for information dissemination and acquisition, particularly during periods of social distancing and stay-at-home orders. On the other hand, anxiety associated with social isolation has the potential to lead to compulsive social media usage and over usage has the potential to further amplify anxiety at the population, community, and individual levels [11,51]. This can lead to a cycle that may be difficult for many to evade [11]. Equally important, overreliance on social media increases the likelihood of receipt of false rumors, hearsay, and inaccurate information [52]. Though the social media source was not collected in the present study, it is plausible that a significant number of participants may have acquired information on COVID-19 from people in their social media networks or from nonauthoritative sources. To minimize psychological distress in the general population, efforts are needed to educate the general public on the necessity of being discriminant with COVID-19 news sources and the benefits of relying on social media posts and information from reliable sources, such as the Ministry of Health of the People’s Republic of China, the Chinese Center for Disease Control and Prevention (CCDC), and the World Health Organization (WHO).

This study had notable limitations and strengths. This study relied heavily on analysis of self-report data, and bias is inherent in self-report data. The cross-sectional nature of the study did not allow for examination of cause–effect relationships. The sample was largely young, and non-probability sampling limited the representativeness of the study population. A one-time email on an institutional listserv and postings on social media platforms for participant recruitment did not allow for capture and quantification of a non-response rate. A single study is unable to identify and include all factors that may be associated with study outcomes. As such, it is probable that there are other factors associated with symptoms of depression and psychological distress that were not included in the study. The study was geographically limited to China. More studies in and outside of China and in developed and underdeveloped countries are needed to fully characterize the adverse impact of the COVID-19 pandemic on psychological well-being. Studies that examine longitudinal changes in psychological well-being during the COVID-19 pandemic are needed as well. Notwithstanding, some generalizations of study findings outside of China are possible given the global widespread and negative impact of the COVID-19 pandemic, particularly among countries with a higher prevalence of COVID-19-related deaths than China (e.g., the Brazil, France, India, Russia, South Africa, Spain, the United Kingdom, and the United States). The sampling strategy successfully recruited a large number of participants (*n* = 2130) in less than 3 weeks. Widely used scales with validated psychometric properties were employed to assess study outcomes.

## 5. Conclusions

Social distancing is a widely used public health approach for population-wide virus-containment of COVID-19. However, reductions in population-wide psychological well-being are inadvertent consequences of social distancing. There is an emerging need to negate factors that increase adverse mental health vulnerabilities during the COVID-19 pandemic.

## Figures and Tables

**Table 1:** Sample characteristics (*n* = 2130).

Variables	Frequency/M, (SD)
**Individual factors**	
Age	28.43 (10.46)
Gender	
Male	962 (45.16)
Female	1168 (54.84)
Education	
≤High school	459 (21.55)
>High school	1671 (78.45)
Marital status	
Married	668 (31.36)
Not married	1462 (68.64)
Income (Chinese currency, CNY ¥)	
¥0–10,000	1209 (56.76)
¥10,001–30,000	264 (12.39)
¥30,001–50,000	247 (11.60)
¥50,001–100,000	235 (11.03)
>¥100,000	175 (8.22)
Self-rated general health	4.35 (0.75)
Practiced social distancing	
No	93 (4.37)
Yes	2037 (95.63)
**Community factors**	
Residential area	
Urban	1014 (47.61)
Non-urban	1116 (52.39)
Living arrangement	
Lived with someone	2059 (96.67)
Lived alone	71 (3.33)
**Environmental factors**	
Amount of time spent daily on social media searching for and reading information on COVID-19	
Never	21 (0.99)
<15 min	261 (12.25)
16–30 min	529 (24.84)
31–45 min	725 (34.04)
56–60 min	348 (16.34)
>1 h	246 (11.55)
Number of confirmed cases of COVID-19 in their city of residence	
None	224 (10.52)
<10	673 (31.60)
10–100	831 (39.01)
101–200	230 (10.80)
201–300	62 (2.91)
>300	110 (5.16)
Confirmed or suspected cases of COVID-19 in personal networks	
No	1847 (86.71)
Yes	283 (13.29)
Mental health outcomes	
Symptoms of depression (CES-D-10)	14.65 (5.49)
Psychological distress (K6)	7.31 (4.71)

**Table 2:** Linear regression predicting symptoms of depression (*n* = 2130).

Predictors	Symptoms of Depression		
Individual factors	b (SE)	*t*	*p*-values
Age	−1.12 (0.68)	−1.64	0.10
Female (ref: male)	0.02 (0.24)	0.12	0.90
High school or above (ref: below High school)	−0.58 (0.29)	−1.96	0.05
Non-married (ref: married)	0.62 (0.45)	1.37	0.17
Income (ref: ≤ ¥10,000, Chinese currency, CNY ¥)			
¥10,001–30,000	0.09 (0.39)	0.23	0.81
¥30,001–50,000	0.21 (0.41)	0.52	0.60
¥50,001–100,000	−0.78 (0.42)	−1.85	0.06
≥¥100,001	−0.77 (0.49)	−1.56	0.12
Self-rated general health	0.04 (0.15)	0.28	0.77
**Practiced social distancing (no)**	**−1.37 (0.59)**	**−2.31**	**0.02**
Community factors			
**Non-urban (ref: urban)**	**0.76 (0.25)**	**3.02**	**0.00**
Lived alone (ref: lived with someone)	0.48 (0.66)	0.73	0.46
Environmental factors			
Amount of time spent daily on social media searching for and reading information on COVID-19	0.00 (0.10)	0.05	0.96
**Number of confirmed cases of COVID-19 in their city of residence**	**0.21 (0.10)**	**2.01**	**0.04**
Confirmed or suspected cases of COVID-19 in personal networks (ref: no)	1.76 (1.04)	1.68	0.09
Model statistics				
F (df)	5.06			
Overall significance	<0.00			
R^2^	0.035			

Significant variables were highlighted in bold.

**Table 3:** Linear regression predicting psychological distress (*n* = 2130).

Predictors	Psychological Distress		
Individual factors	b (SE)	*t*	*p*-values
Age (log transformed)	−0.88 (0.58)	−1.52	0.12
**Female (ref: male)**	**0.63 (0.20)**	**3.09**	**0.00**
**High school or above (ref: below High school)**	**−0.53 (0.25)**	**−2.13**	**0.03**
**Non-married (ref: married)**	**0.94 (0.39)**	**2.43**	**0.01**
Income (ref: ≤ ¥10,000, Chinese currency, CNY ¥)			
¥10,001–30,000	0.41 (0.34)	1.23	0.22
¥30,001–50,000	0.42 (0.35)	1.19	0.23
¥50,001–100,000	0.08 (0.36)	0.22	0.82
≥¥100,001	0.07 (0.42)	0.17	0.86
Self-rated general health	0.05 (0.13)	0.42	0.67
**Practiced social distancing (no)**	**1.38 (0.21)**	**2.73**	**0.00**
Community factors			
Non-Urban (ref: urban)	0.25 (0.21)	0.16	0.24
Lived alone (ref: lived with someone)	−2.26 (0.56)	−0.47	0.63
Environmental factors			
**Amount of time spent daily on social media searching for and reading information on COVID-19**	**0.32 (0.08)**	**3.73**	**0.00**
**Number of confirmed cases of COVID-19 in their city of residence**	**0.23 (0.09)**	**2.57**	**0.01**
**Confirmed or suspected cases of COVID-19 in personal networks (ref: no)**	**2.96 (0.89)**	**3.31**	**0.00**
Model statistics			
F (df)	5.19		
Overall significance	<0.00		
R^2^	0.036		

Significant variables were highlighted in bold.

## Data Availability

Data is available on request to the corresponding author (F.Y.).
